# Protocol of a meta-analysis: Clinical efficacy and complications of short versus long fusion for the treatment of degenerative scoliosis

**DOI:** 10.1097/MD.0000000000018845

**Published:** 2020-01-17

**Authors:** Wanjun Liu, Jian Sun, Yao Wu, Liqi Yang

**Affiliations:** The Second Affiliated Hospital of Anhui Medical University, Hefei, Anhui, China.

**Keywords:** degenerative scoliosis, fusion, meta-analysis, surgery

## Abstract

**Background:**

The purpose of this study was to evaluate the effectiveness and safety of long fusion (LF) versus short fusion (SF) for the treatment of degenerative scoliosis (DS).

**Methods:**

We will search MEDLINE, EMBASE, PubMed, the Cochrane Library, and Web of Science to collect the randomized and non-randomized controlled studies that compared LF with SF in the treatment of DS from inception to June 1, 2019. The quality of the included studies will be assessed by 2 evaluation members according to the Cochrane collaboration network standard or the Newcastle–Ottawa Scale. The included studies will be analyzed using RevMan 5 (version 5.3.3).

**Results and Conclusion:**

The study will compare the efficacy and safety of LF and SF in the treatment of DS and provide more reliable, evidence-based data for clinical decision making.

**PROSPERO registration number:**

CRD42019137646.

## 1 Introduction

Degenerative scoliosis (DS), which is more common in the elderly, is a deformity of the coronal Cobb angle ≥ 10° caused by the degeneration of individuals with mature bone development.^[1]^ DS is a complex spinal disease, and its exact pathology is unclear.^[2]^ In addition, its clinical symptoms vary, including lumbago backache, lower limb pain, and trunk unbalance.^[3]^ At present, the primary treatment for DS in clinical practice is surgery, which mainly includes decompression and fusion. Its purpose is to relieve lumbago and neurogenic pain, correct the deformity, and reconstruct the balance of the spine.^[4]^ However, there is a lack of consensus on the length of fusion in the surgical treatment of DS.^[5]^ According to the definition published in previous studies, short fusion (SF) refers to cases where either the average number of fusion segments is less than 3 or the fusion segments are within the upper and lower vertebrae of scoliosis, whereas long fusion (LF) includes cases where the average number of fusion segments is equal or greater than 3 or the fusion segments reach or exceed the upper and lower vertebrae of scoliosis.^[6,7]^ The purpose of our study was to compare the efficacy and safety of LF and SF in the treatment of DS and to provide more reliable, evidence-based data for clinical decision making.

## 2 Methods

### 2.1 Standards

This protocol will be performed based on the Preferred Reporting Items for Systematic Reviews and Meta-Analyses Protocols guidelines.

### 2.2 Ethical issues

Ethical approval is not required as this study is based on aggregate data and will not involve humans.

### 2.3 Registration

The protocol has been registered in the PROSPERO and the registration number is CRD42019137646.

### 2.4 Inclusion criteria

Clinical studies including prospective and retrospective observational studies (cohort studies, case–control studies, and cross-sectional studies) will be considered eligible. In addition, we will only include articles published in English. All studies should assess at least one of the following parameters: surgical duration, hospital stays, blood loss, Cobb angle, coronal C7 plumb, visual analog scale scores, Oswestry Disability Index, and complications.

### 2.5 Search strategy

We will search MEDLINE, EMBASE, PubMed, the Cochrane Library, and Web of Science from inception to June 1, 2019. The search terms included (“degenerative scoliosis” or “degenerative spinal deformity” or “coronal imbalance”) and (“fusion” or “internal fixation” or “curve correction”).

### 2.6 Data analysis and statistical methods

All the data will be subjected to meta-analysis using RevMan 5 (version 5.3.3, Cochrane, London, UK). Statistical heterogeneity will be assessed by chi-square and *I*^2^ tests. If the *I*^2^ value is >50%, the data will be considered to be significantly heterogeneous. Continuous data will be represented by mean differences and 95% confidence intervals whereas dichotomous data will be represented by odd ratios and 95% confidence intervals. A *P* value of <0.05 will be considered statistically significant.

### 2.7 Quality assessment

Two researchers will independently evaluate the quality of the literature. Studies will be evaluated using the Cochrane risk of bias tool and Newcastle–Ottawa scale.

## 3 Discussion

DS is a common degenerative disease of the spine and often requires surgical treatment.^[8]^ Researchers have suggested that severe or progressive deformities, progressive aggravation of neurological symptoms, and ineffective conservative treatment are the main surgical indications.^[9]^ Although decompression alone may be effective in relieving neurological symptoms, it may lead to further spinal instability.^[10]^ Therefore, most surgeons recommend decompression combined with fusion for the treatment of DS.^[11]^ However, there is no uniform standard for the length of fusion segments. The study will compare the efficacy and safety of LF and SF in the treatment of DS and provide more reliable, evidence-based data for clinical decision making.

## Author contributions

**Conceptualization:** Wanjun Liu, Jian Sun, Yao Wu.

**Data curation:** Wanjun Liu, Jian Sun.

**Formal analysis:** Wanjun Liu, Jian Sun, Yao Wu.

**Funding acquisition:** Wanjun Liu, Jian Sun, Liqi Yang.

**Methodology:** Wanjun Liu, Jian Sun.

**Supervision:** Liqi Yang.

**Writing – original draft:** Wanjun Liu, Jian Sun.

**Writing – review & editing:** Wanjun Liu, Jian Sun, Liqi Yang.
